# Device-Oriented CFD Comparison of Rectangular and Circular Microchannels with Single and Double Asymmetric Stenoses Under Identical Operating Conditions

**DOI:** 10.3390/bioengineering12121313

**Published:** 2025-11-30

**Authors:** Mesude Avcı

**Affiliations:** Department of Chemical Engineering, Cumhuriyet University, 58140 Sivas, Turkey; mesude@cumhuriyet.edu.tr; Tel.: +90-531-024-5513

**Keywords:** asymmetric stenosis, computational fluid dynamics (CFD), microfluidic channels, wall shear stress, hemodynamics, in vitro vascular models, medical device design, device-oriented comparison

## Abstract

Microchannels can create disturbed flow patterns by altering pressure gradients, shear forces, and flow symmetry, which are essential in the design of microfluidic devices and, hence, blood-contacting devices. The effect of asymmetric stenosis on pressure, wall shear stress, and velocity in rectangular and circular microchannels with same operating conditions was analyzed in this study using three-dimensional (3D) steady laminar computational fluid dynamics (CFD) simulations. Asymmetric flow patterns induced by asymmetric stenosis are of particular importance and remain underexplored, especially in the context of multiple constrictions. This is, to our knowledge, is the first systematic CFD comparison of multiple asymmetric stenoses in circular microchannels directly contrasted with rectangular and single-stenosis cases under identical settings. Several parameters, such as wall shear stress (WSS), pressure, and velocity distributions, were analyzed in various stenotic and non-stenotic geometries. These microchannel models, while not reflecting real blood vessels themselves nor exhibiting wall compliance, pulsatility, or non-Newtonian rheology, replicate important mechanical characteristics of stenosis-mediated flow disturbance. Single and multiple asymmetric stenoses create flow patterns that are similar to those of vascular pathologies. For this reason, these channels should be considered as simplified device-scale models of vascular phenomena as opposed to realistic, in vitro vascular models. The results showed that asymmetric stenosis creates asymmetric velocity peaks and elevated WSS, which are more evident in the case of circular configurations with double asymmetric stenosis. The findings will help design microfluidic devices that mimic unstable flow characteristics that occur in stenotic conditions, and assist in testing clinical devices. In this study, two fabrication-ready microchannel designs under fixed operating conditions (identical inlet velocity and fluid properties) that reflect common microfluidic use were compared. Consequently, all pressure, velocity, and WSS outcomes are interpreted as device-scale responses under fixed velocity, rather than a fundamental isolation of cross-section shape, which would require matched hydraulic diameters or flow rates. This study is explicitly device-oriented, representing a fixed operating point rather than a strict geometric isolation. Accordingly, the results are also expressed with dimensionless loss coefficients (Ktot and Klocal) to enable scale-independent, device-level comparison.

## 1. Introduction

Stenosis, the narrowing of blood vessels, and its hemodynamic effects have been studied commonly in large arteries such as the aorta, carotid, and coronary vessels. Stenosis can also occur in much smaller vessels, which have diameters of only a few hundred microns. Therefore, disturbed flows caused by stenosis are a critical factor, and simplified microfluidic models are very appropriate to investigate these hemodynamic mechanisms. At such micro-scales, a controlled in vitro microfluidic model is necessary to investigate geometry-driven hemodynamics independent of wall compliance, pulsatility, and the hematocrit-dependent rheology. In contrast to arterial stenoses, which, in general, have deformable walls that expand and contract with pressure, exhibit physiological pulsatility, and contain cell-rich, hematocrit-dependent rheology, microfluidic channels are designed with rigid walls, uniform and well-regulated inflow, and well-defined geometries [1,2]. These parameters enable geometry-driven effects, such as velocity jetting, increases in wall shear stress (WSS), and step-like pressure losses [3,4,5], to be controlled and reproduced. Despite these microscale-specific features, the current microfluidic channels purposefully utilize rigid walls, steady laminar inlet conditions, and a Newtonian fluid model to separate geometry-driven hemodynamics in a simplified device-driven manner and not to capture the complexity of in vivo hemodynamics or disturbed-flow behavior present in physiology-based vessels [6]. Therefore, the current work intentionally compares cross-section shape and stenosis number under identical conditions involving asymmetric and multiple constrictions within rectangular and circular microchannels of identical size and severity. This work intentionally targets a steady, laminar microfluidic regime (low Re, rigid walls, uniform inlet), commonly used in in vitro thrombosis and hemocompatibility assays rather than pulsatile arterial flows. In this regime, geometry-driven losses and wall shear stress (WSS) peaks dominate and can be precisely tuned to reproduce controlled shear conditions relevant to microfluidic device design [7,8,9].

Study Framing and Scope: The present study is explicitly device-oriented. Rectangular and circular microchannel designs were selected as fabrication-ready configurations and were operated under fixed, identical inlet velocity and fluid properties, replicating the syringe-pump conditions commonly used in microfluidic experiments [5,9]. Accordingly, the results represent device-oriented performance under the same operating point, rather than a fundamental isolation of cross-sectional geometry. To facilitate interpretation across designs, pressure losses were expressed as a dimensionless total loss coefficient (Ktot), and the stenosis-induced incremental local loss (Klocal) was computed within each geometry, consistent with laminar microchannel practice [10,11].

Hemodynamic research has been extensively focused on flow disorders caused by idealized stenotic geometries, particularly in microfluidic devices, which are designed to mimic vascular-like conditions. Due to the common clinical importance of vascular stenosis, hemodynamic research has increased significantly in recent decades. In vivo, experimental methods are considered the most realistic means of acquiring physiological data and remain fundamental to establishing a sufficient understanding of basic biophysical phenomena. However, performing such experiments in animals does not fully replicate human physiology and disease. Moreover, those experiments are expensive, time-consuming, not reproducible, and have serious ethical concerns [12,13,14]. Consequently, the development of engineered in vitro biomodels, such as microfluidic channels, has gained much attention to address these limitations in a controlled and reproducible approach. These methodologies are frequently employed to validate numerical studies and complement the results of experimental investigations [1]. Such numerical studies include computational fluid dynamics (CFD), which is known as a valuable tool to study hemodynamics, and also has advantages when compared to experimental analysis, such as flexibility and efficiency.

CFD has been widely used to examine vascular flow disturbances by modeling small-scale and high-efficiency microfluidic devices, which offer a distinctive and adaptable design [15,16,17,18]. These microfluidic environments, usually referred to as ‘lab on a chip’ systems, facilitate investigating the hemodynamic forces on vascular and hematological cell behavior through their fluidic and physical properties [19,20,21]. Additionally, they facilitate the examination of micro-scale processes by controlling agonist exposure and ligand presentation [3,22,23,24], and thus offer an adaptable environment to study cellular responses under different shear conditions. Specific materials can be used to design microfluidic chips that reproduce the shear profiles of local regions, which are representative of vascular or medical device environments. The capability to computationally determine and precisely control fluid dynamics in microfluidic devices makes these excellent tools for studying and mimicking vascular-like flow conditions that arise at determined hemodynamic shear conditions [4,25]. For example, microfluidic devices with contractions or humps have been developed to simulate flow disturbances in microchannel stenosis models [21,22,26]. CFD is also widely used to model such microfluidic flow conditions before conducting experiments [26,27]. However, the effect of shear rates in disturbed flows, such as those caused by stenosis, remains uncertain. In particular, the influence of microfluidic boundary conditions on hemodynamic parameters associated with flow disruption, including the peak shear rate, peak wall shear stress (WSS), and shear rate gradient at the stenotic (narrowing) zone, remains inadequately described [22,27,28].

Symmetric (also known as concentric) stenosis has been widely studied, which shows its importance in hemodynamic investigations [15,17,29]. Jung et al. examined the blood flow behavior in vessels with 50%, 57%, and 75% stenosis and determined peak velocity in all stenosis models [16]. Nadeem et al. [30] used a mathematical model and simulated 50–80% stenosis, resulting in increased velocity and pressure drop with advancing stenosis severity. El Kot et al. [31] solved multiple symmetric stenosis numerically and found increased blood velocity. Moreover, Ali et al. [32] solved a mathematical model numerically to explore the blood flow in a symmetric stenosed artery and showed that maximum wall shear stress occurred at the critical height of the stenosis. Additionally, multiple stenosis associated with spiral blood flow, which can be caused by curvatures of the vessel or cardiac movement, have been investigated [33,34,35]. Blood flow and its spiral components are essential and have been investigated through symmetric stenosed arteries with varying area reductions for multiple stenoses [36]. The findings indicated velocity peaks at the stenosis in both single and double stenosis, and it was found that fluid properties increased with the increase in area reduction.

Other parameters, such as asymmetry, may be considered for a more precise evaluation of disturbances occurring in flow beyond stenosis severity, since they can have important biomechanical implications [2]. Asymmetry is a significant parameter, as its alterations cause asymmetric flow patterns. Furthermore, non-planarity caused by eccentricity (i.e., lateral asymmetry) can increase helical flow, which was caused by stenosis curvatures and non-planarity [2]. For example, it was seen that eccentricity (i.e., lateral asymmetry) increased the turbulence intensity [37]. Similarly, significant differences on shear stress distribution and magnitude were observed between concentric and asymmetric stenosis cases [38]. When eccentric (i.e., lateral asymmetry) stenosis was compared to its concentric counterpart, the maximum wall shear stress at the stenosis throat decreased largely at the outer wall.

When the existing literature is analyzed, to the best of our knowledge, no previous study has systematically compared the effect of geometrical shape on stenosed and non-stenosed channels, and also the impact of stenosis numbers on microchannels with different geometrical shapes. Microfluidic devices with a single stenosis and with multiple concentric stenoses have been studied previously [5,26,34,35], but no work has modeled multiple asymmetric stenoses. Furthermore, while stenosis configurations have been studied, they have focused predominantly on concentric stenosis with only a single constriction, and usually in one type of channel shape. In contrast, the current study addresses this gap by systematically investigating single and double asymmetric stenoses in both rectangular and circular microchannels of the same size and the same stenosis severity. The novelty of this study lies in evaluating hemodynamic differences as consequences of geometry and setup, rather than numerical or modeling artifacts, by combining the effects of asymmetry and multiplicity on axial velocity, pressure drop, and wall shear stress. Therefore, this study also models the effect of multiple asymmetric stenoses for the first time. Unlike earlier studies that considered a single constriction or one channel shape, this work systematically compares multiple asymmetric stenoses in circular and rectangular microchannels under identical flow conditions, providing new design insights for in vitro microfluidic assays [7,9]. This study has several distinct objectives as explained below:

As a starting point, a rectangular-shaped microfluidic device with a single asymmetric stenosis, as modeled by Wu et al. [39], was created, and the results were validated against their results. Then, a non-stenosed rectangular microchannel was created to examine the effect of stenosis on rectangular microchannels, and its results were compared with those of the stenosed channel. The impact of hemodynamic parameters, including wall shear stress (WSS), pressure, and velocity, on stenosed and non-stenosed asymmetric stenosis was investigated.

Second, this study aims to clarify the debate over the use of rectangular or circular microchannels to better represent medical device geometries, since there are different aspects to consider when using which shape of microchannel to represent clinical devices better. Previous studies stated that microchannels used for diagnostic applications and the treatment of several chronic diseases have rectangular geometries [29,40]. On the other hand, most studies investigated stenosis on circular models because they are considered closer to vascular structures [5,26,34,35]. Therefore, for a better evaluation of this argument, circular microfluidic devices without stenosis and with single asymmetric stenosis, which have identical dimensions with rectangular microchannels, were also constructed. Comparison and evaluation of rectangular and circular geometries with no stenosis and with a single asymmetric stenosis was easily performed.

Finally, as discussed above, multiple stenoses are important because they may represent spiral-like blood flow, which occurs due to vessel curvature or cardiac movement. Multiple symmetric (concentric) stenosis has been studied previously, but studies on multiple asymmetric stenosis are limited. Therefore, multiple asymmetric stenosis in circular microchannels was also investigated in this work. Overall, all models (rectangular microchannels without and with single asymmetric stenosis, circular microchannels without, with a single, and with double asymmetric stenoses) were systematically compared. These results show the effect of geometrical shape on both stenosed and non-stenosed channels. Also impact of stenosis numbers on microchannels with different geometrical shapes was investigated. Contributions of this study are (i) it is a first CFD study, which shows systematical analysis of multiple asymmetric stenosis in circular microchannels, (ii) it provides comparisons for hemodynamic behavior between circular and rectangular geometries, (iii) it shows a clear evaluation for pressure loss, velocity peaks, and WSS increase due to stenosis, (iv) it enlightens the effects of stenosis number and asymmetry on disturbed flow. Although Newtonian and idealized models have been widely employed in previous CFD studies, an innovation of the present work is simultaneously combining and systematizing the effect of asymmetry and multiplicity for both the circular and rectangular geometries of the same dimensions. This work may offer a reproducible benchmark between simplified microfluidic models and the design of physiologically realistic in vitro vascular systems and blood-contact medical devices.

## 2. Materials and Methods

### 2.1. Geometry Modeling

**Figure 1:**
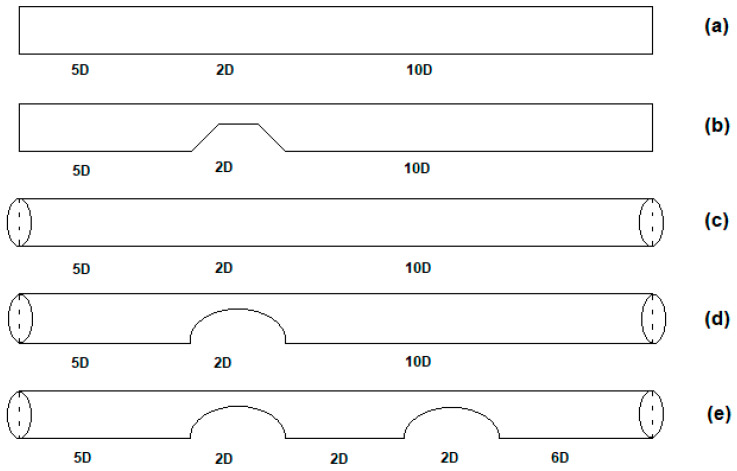
Schematic representations of modeled microchannel geometries used in CFD simulations. All dimensions are given in μm. Configurations include: (**a**) rectangular microchannel without stenosis, (**b**) rectangular microchannel with single asymmetric stenosis, (**c**) circular microchannel without stenosis, (**d**) circular microchannel with single asymmetric stenosis, and (**e**) circular microchannel with double asymmetric stenosis. All geometries share an identical inlet diameter and length, with 80% area reduction applied in all stenotic models.

Microfluidics is a miniaturized approach with considerable advantages in regulating hemodynamic parameters at the micro-scale [3,22,23,24]. In this study, asymmetric stenosis was systematically investigated in idealized microchannels with different shapes in the case of no stenosis and with single and double asymmetric stenosis. The variations in velocity distribution, pressure drop, and wall shear stress were examined. Thus, a total of five models were analyzed: two rectangular microchannels (one with no stenosis and one with a single asymmetric stenosis) and three circular microchannels (one with no stenosis, one with a single asymmetric stenosis, and one with double asymmetric stenosis) (Figure 1).

Modeling was started by recreating the rectangular microfluidic device, which has a single asymmetric stenosis, of Wu et al. [39] as a 3D model with the exact dimensions, and the results were compared with theirs using ANSYS Fluent 18.2. Then, this channel was used as a reference to compare with other models, which have different shapes and numbers of stenosis. The axial length, width, and height (diameter for circular) of the microfluidic device’s cross-section were 1607.4 µm, 130 µm, and 100 µm, respectively, which are the dimensions that Wu et al. [39] used in their model. The channel height (in rectangular) or diameter (in circular) models were extruded along the z-axis in a 3D geometry and no-slip boundary conditions were applied to all channel walls. The rectangular inlet (130 × 100 µm) and the circular inlet (D = 100 μm) were not matched in cross-sectional area or hydraulic diameter. The rectangular section has A = 1.30 × 10^4^ μm^2^ with hydraulic diameter D_h_ = 2ab/(a + b) = 113 μm, whereas the circular section has A = πD^2^/4 = 7.85 × 10^3^ μm^2^ with D_h_ = 100 μm. To ensure a fair cross-geometry comparison without enforcing equal area, all cases were run under identical inlet velocity, fluid properties (ρ, μ), inlet profile (uniform), outlet condition (outflow), wall condition (rigid, no-slip), numerical settings (laminar model, SIMPLE coupling, second-order schemes), convergence criterion (scaled residuals < 1 × 10^−6^), total domain lengths, stenosis severity (80% area reduction), and stenosis placement (single at 5D; double with 2D spacing). Under these fixed conditions, observed differences can be attributed to geometry. This configuration constitutes a device-oriented fixed-velocity comparison; it is not a strict geometric isolation, which would require matched hydraulic diameters and/or flow rates. This design choice aligns with prior evidence that laminar hydraulic resistance depends on cross-sectional shape in microchannels [10]. The asymmetric stenosis was placed at 5D for the single stenosis models (rectangular and circular), which was directly obtained from Wu et al. [39]. However, unlike Wu et al. [39], the downstream length was extended from 5D to 10D to minimize outlet boundary effects. In the double stenosis model, the first stenosis was placed at 5D according to Wu et al. [39] and the second stenosis was placed at one stenosis length (2D) after the first constriction. The distance between the two stenoses was set as two diameters and held constant to isolate geometric effects and number-of-stenosis effects from all other variables. The placement of the second stenosis was arbitrarily defined since Wu et al. [39] did not model a double stenosis case. Between all five models, the overall lengths, diameters, and stenosis geometries were maintained identically to perform a correct comparison between models. The asymmetric constriction means a narrowing displaced from the centerline, which is commonly referred to as “eccentric” in vascular research to define stenosis shifted from the center. The cross-sectional area of the throat was set to present an 80% reduction in the sectional area. The asymmetry was obtained by moving the throat center away from the channel central axis. The same geometric definition was applied for all stenosed cases so that comparisons would differentiate flow effects from geometry and the number of stenosis. The stenosis severity was used as an 80% area reduction, which geometrically means approximately a 55% diameter reduction. For clinical context, stenosis of at least 80% is treated as a severe category in the carotid literature, especially for in-stent restenosis, where duplex ultrasound criteria and outcome analyses commonly use the 80% threshold” [41,42]. Similarly, reviews of asymptomatic carotid stenosis also summarize consideration windows of approximately 60% to 80% [43]. Here, 80% area reduction is used largely for the sake of consistency with Wu et al. [39], and to produce a consistent and pronounced constriction, which is independent of geometry and the number of stenoses under identical boundary conditions. This clarifies the geometric description, which also shows that models simulated here stay simplified microfluidic channels rather than physiological vessels. The simplified rectangle and circle shapes were selected to focus on hemodynamics with a specific geometry and number of stenoses in a controlled manner that is also repeatable. These reduced-order models are frequently used in microfluidics studies, which do not aim to replicate the complete physiological complexity of vascular structures but aim to provide benchmark test cases for systematic analysis. Moreover, the rigid-wall assumption, in the absence of fluid–structure interaction, was used similarly to standard microfluidic device designs, which allows reproducible flow distortion measurements without additional structural uncertainties. The geometric definition of asymmetry (e) was the offset of the stenosis throat center from the microchannel centerline, normalized by the channel radius (R) (e = offset/R).

The blood was assumed to be laminar, incompressible, and Newtonian, with a density of 1060 kg/m^3^ and a viscosity of 0.003450 Pa.s, which is a commonly used and valid assumption when modeling microchannels [15,44,45]. Although blood shows shear-thinning behavior at low shear rates, the characteristic shear rates are typically much higher in microfluidic channels. To verify this, both the volume-weighted average (<γ>) and the maximum shear rate (γ_max) over the fluid domain were computed for each configuration. As shown in Table 1, all cases have shear rates much higher than 500 s^−1^, which is a widely accepted threshold above which blood rheology can be reasonably approximated as Newtonian [46,47]. To further justify the Newtonian assumption quantitatively, shear rates were compared with a commonly accepted threshold of 500 s^−1^ for microchannel studies. For the five tested cases (Table 1), volume-averaged shear rates were observed to be between 908.86 and 1826.13 s^−1^, while the maximum shear rates vary in the range of 2.32 × 10^4^–6.79 × 10^4^ s^−1^. That is, the average values are 1.8–3.7 times larger than 500 s^−1^, and maxima are about 46–136 times larger. The flow is well beyond 500 s^−1^, under a high shear regime, such that the shear-thinning corrections can be neglected, and Newtonian model utilization becomes quantitatively adequate for the present microchannel conditions. Experimental evidence was also provided to confirm Newtonian-like shear flow of blood under such high shear regimes [48]. Therefore, the Newtonian assumption was used in this study, which helps to reduce computational cost without affecting velocity and wall shear stress predictions.

### 2.2. Computational Procedures

**Figure 2:**
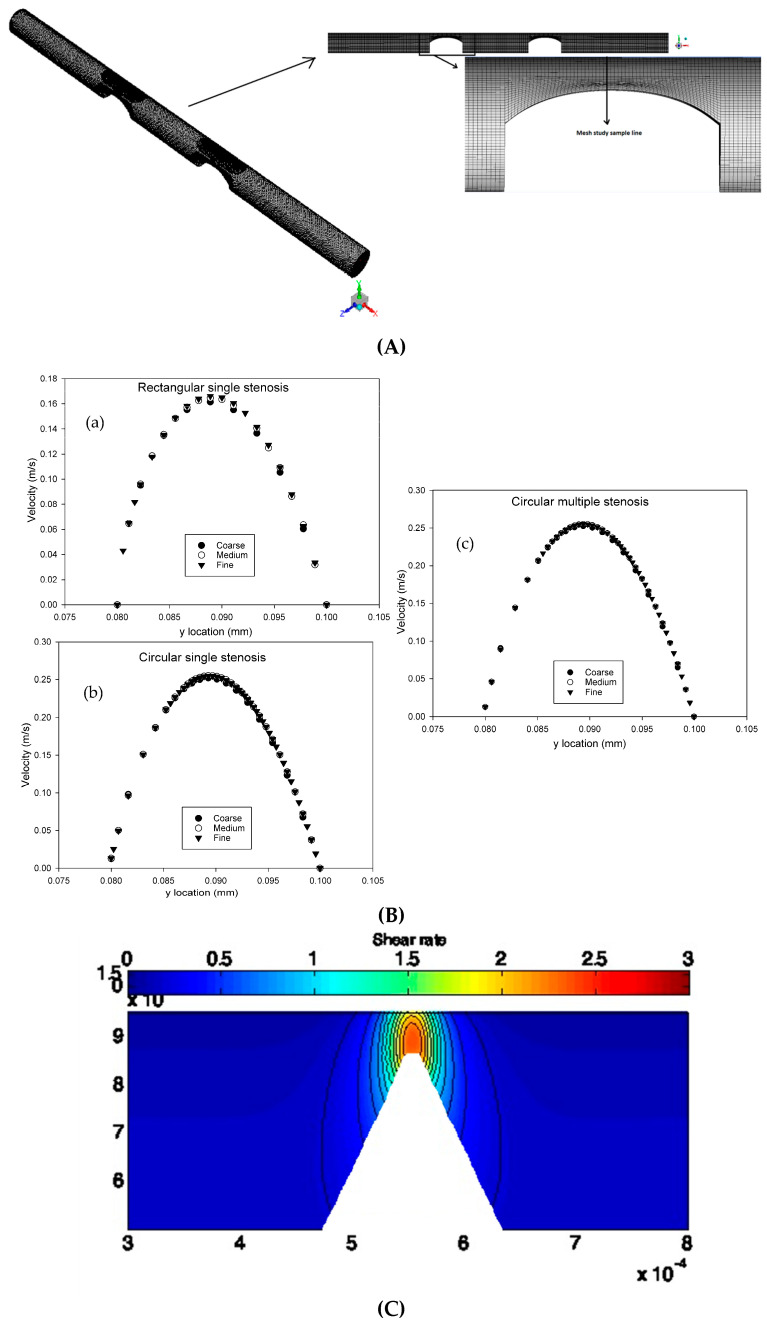
(**A**) Structured 3D hexahedral mesh of the microchannel. Left: isometric 3D view showing the domain depth (z-extent). Top: side view of the channel geometry; Right: zoom near the one-sided throat with the mesh-study sample line used in the grid-independence analysis. (**B**) Grid independence analysis showing mean velocity profiles along a vertical line located 65 µm from the channel wall in: (**a**) rectangular microchannel with single asymmetric stenosis, (**b**) circular microchannel with single asymmetric stenosis, (**c**) circular microchannel with double asymmetric stenosis. Velocity convergence across mesh sizes demonstrates solution accuracy. (**C**). Shear rate distribution in the XY plane at z = 30 μm for the rectangular microchannel with single asymmetric stenosis. The results validate consistency with findings from Wu et al. [38].

Three-dimensional, steady, laminar, incompressible simulations were performed in ANSYS Fluent (v18.2) with second-order spatial discretization; convergence was indicated when scaled residuals were less than 1 × 10^−6^. Five cases with different geometries (shown in Figure 1) were meshed with structural hexahedral elements distributed non-uniformly. The grid nodes near the channel wall and the stenosis were more densely populated than in other regions, thus enabling the simulation to capture and recognize the flow patterns effectively. The fine meshes used for the final simulations contained 478,509 cells for the rectangular no-stenosis model, 814,424 cells for the rectangular single-stenosis model, 549,623 cells for the circular no-stenosis model, 867,637 cells for the circular single-stenosis model, and 1,225,559 cells for the circular double-stenosis model. A representative view of the computational mesh is shown in Figure 2A. To ensure numerical robustness, mesh independence tests were also performed, and their results are presented in Section 2.3.

After meshes were created, the modeling was performed in Fluent to solve the incompressible Navier–Stokes equations. The finite volume-based Fluent simulator was used to model five different microfluidic models and solve the governing equations in the flow fields. The inlet velocity was used as 0.0205 m/s, which was used in the work of Wu et al. [39]. The specified boundary conditions for the idealized microchannel models were determined as the velocity-inlet at the domain inlet, an outflow at the domain outlet, and the no-slip condition for the nozzle walls. Pressure-loss comparisons are presented at a fixed uniform inlet velocity with the rectangular and circular inlets not matched by cross-sectional area or hydraulic diameter. Under such conditions, total pressure loss is a measure of both cross-section shape and stenosis-induced constriction or expansion losses rather than the fully developed, equal-area, fixed-flow rate case [10,11]. Fluid flow was assumed to be laminar and steady. Reynolds number was evaluated using the hydraulic diameter Dh as Re = ρUDh/μ with ρ = 1060 kg m^−3^, μ = 3.45 × 10^−3^ Pas, and the inlet velocity U = 0.0205 m^s−1^. For the rectangular inlet (130 × 100 µm), D_h_ = 2ab/(a + b) = 113 µm giving Re = 0.71. For the circular inlet (D = 100 µm), D_h_ = 100 μm giving Re = 0.63. For completeness, the volumetric flow rate was computed as Q = AxU. Under the fixed inlet velocity (U = 0.0205 m/s), Q was 16.0 μL/min for the rectangular microchannel (A = 1.30 × 10^4^ μm^2^) and 9.66 μL/min for the circular microchannel (A = 7.85 × 10^3^ μm^2^), corresponding to a 66% higher flow rate in the rectangular case (Table 2).

Since same inlet conditions and fluid properties are used in each geometry type, these values are valid for all corresponding cases and confirms a strictly laminar regime. In Ansys Fluent, the second-order upwind scheme was applied to discretize the pressure and momentum equations since the second-order considers two upstream points and gives more accurate results when compared to the first-order scheme [49]. Steady-state flow was used rather than pulsatile flow since the current modeled systems are microfluidic devices with steady inlet boundary conditions rather than pulsatility that exists in the body. This approach allows comparison of geometrical effects in a controlled manner, without the additional variation in their effect due to transient flow oscillations, and is in line with previous computational fluid dynamics (CFD) microchannel flow studies [39,49]. The SIMPLE algorithm was utilized for pressure-velocity coupling, which has a lower computational cost and performs well in steady simulations [49]. Convergence was declared when the scaled residuals for continuity and x-, y-, z-momentum fell below 1 × 10^−6^ before reporting results. In conjunction with the residual-driven requirement, certain key physical parameters (outlet velocity, pressure at a fixed monitoring point, and area-weighted wall shear stress) were checked to ensure physical convergence. Dimensionless normalization: To enable a fair and scale-independent cross-geometry comparison without forcing equal cross-sectional area or hydraulic diameter, total pressure losses were reported as a dimensionless loss coefficient (K_tot_) as;

(1) $$K_{tot}=\frac{∆P}{0.5\rho U^{2}}$$

where ΔP is pressure loss, ρ is the density and U is the inlet velocity. For stenosed configurations, the incremental local loss (K_local_) was also evaluated as;

(2) $$K_{local}=K_{tot,stenosed}-K_{tot,no\text{-}stenosis}$$

where K_local_ is incremental local loss, K_tot,stenosed_ loss coefficient in stenosed channel and K_tot,no-stenosis_ is the loss coefficient without stenosis. This normalization follows the shape-dependent hydraulic resistance framework for microchannels and measured inlet/contraction loss data [10,11]. K local values were calculated within the same geometry, isolating the additional contribution of asymmetric constrictions.

**Recirculation length measurement:** For stenosed cases, a near-wall polyline was placed on the open side at the mid-plane (offset 3 µm from the wall), starting 0.1 D downstream of the throat and extending to 1–2 D upstream of the outlet. The axial velocity (u_x_) was sampled along this line and plotted versus x. The reattachment point was defined as the first downstream location where u_x_ changed sign from negative to non-negative; the recirculation length was the axial distance from the throat exit to this point. Values are reported in mm and normalized by D. For no-stenosis baselines, no reversed flow was observed (L_rec_ = 0).

### 2.3. Validation of the Numerical Model

Validation was performed in two steps: (i) mesh independence analysis to check the consistency, and (ii) comparison of simulation results with previous data. A grid-independence test, which entails computing the solution on successively finer grids, was conducted to ascertain the sensitivity of the numerical solutions. The grid independence test was performed individually for each geometry under consideration. The results from the three geometries, namely the rectangular and circular models with single stenosis and the circular model with double stenosis, were presented for brevity. Models with three different mesh sizes (coarse, medium, and fine) were run for each geometry considered. Then, velocity profiles were analyzed along a 20 µm vertical sample line, located 65 µm away from the channel wall in the z-direction and connected between the apex of the stenosis and the ceilings of the microfluidic channels (shown in Figure 2A). Mean velocity magnitude results are shown in Figure 2B. The grid independence test results for three microchannels indicated no significant differences in the velocity profiles for coarse, medium, and fine grid sizes.

In addition to velocity profiles, mesh convergence was also carried out by comparing medium and fine meshes for global (pressure drop, ΔP) and local (peak WSS) parameters (Table 3). The ΔP change was always under 2% in all the cases, indicating that the convergence of global hemodynamic quantities was good. On the other hand, peak WSS presented higher sensitivity to the mesh with the highest difference among all single cases, ~23%, in the rectangular single stenosis case. This rather large discrepancy is in line with the widely known sensitivity of near-wall stresses to grid resolution and therefore confirms the choice of the fine mesh for all subsequent simulations.

Mesh Convergence via Grid Convergence Index (GCI): To quantitatively assess the mesh-independence of the numerical results, a formal Grid Convergence Index (GCI) analysis was performed following Roache [50]. Three systematically refined meshes (coarse, medium, and fine) were generated for representative rectangular and circular microchannel models. The refinement ratio r, observed order of accuracy p, extrapolated value ϕ_ext_, and fine-grid convergence index GCI_fine_ were evaluated using the following relations:

(3) $$r={\left(\frac{N_{f}}{N_{m}}\right)}^{1/3},p=\frac{ln\left(\frac{\phi_{c}-\phi_{m}}{\phi_{m}-\phi_{f}}\right)}{ln\left(r\right)},\;\phi_{ext}=\phi_{f}+\frac{\Phi_{c}-\Phi_{m}}{r^{p}-1},\;{GCI}_{fine}=F_{s}\frac{\left|\Phi_{f}-\Phi_{m}\right|}{\left|\Phi_{f}\right|\left(r^{p}-1\right)}$$

where N_c_, N_m_, N_f_ were number of cells in the coarse, medium, and fine meshes, respectively. ϕ_c_, ϕ_m_, ϕ_f_ were the computed quantity (ΔP) on each mesh level, r was grid refinement ratio derived from cell counts, p was observed order of convergence, ϕ_ext_ was the Richardson-extrapolated value approximating the asymptotic exact solution, GCI_fine_ was fine-grid convergence index, representing the numerical uncertainty of the fine mesh, F_s_ = 1.25 (safety factor recommended by Roache [50]). In this study, the formal GCI was evaluated for the global pressure drop (ΔP), which shows monotonic convergence and best reflects solution accuracy in a global sense. The resulting fine-grid GCI for ΔP was below 5% for all representative cases, confirming that the fine mesh provides sufficient spatial resolution for all subsequent simulations (Table 4).

The second step was to validate the rectangular-shaped microchannel with a single asymmetric stenosis by comparing it with the microchannel of Wu. et al. [39]. Figure 2C illustrates the shear rate distribution in the XY plane at z = 30 μm. When the shear rate is compared to the results of Wu et al. [39], the shear rate values exhibit similar spatial behavior throughout the plane and are qualitatively in good agreement. At the matched plane (z = 30 μm), the maximum shear rate falls within the 3.0 (red) band on the 0–3.0 scale, which is the same band reported by Wu et al. [39]. The adjacent 2–3 contour lines around the throat is very similar in both figures and positions on different color change by no more than one contour interval. This indicates close quantitative agreement in addition to visual similarity. This demonstrates that the CFD setup can reliably simulate the key flow characteristics occurring in stenotic conditions of microchannels, even though the model does not represent a physiological vessel.

## 3. Results and Discussion

All results reported below were obtained on the fine mesh; mesh-independence is documented in Table 3 and Table 4.

### 3.1. Velocity Profiles and Distributions

**Figure 3:**
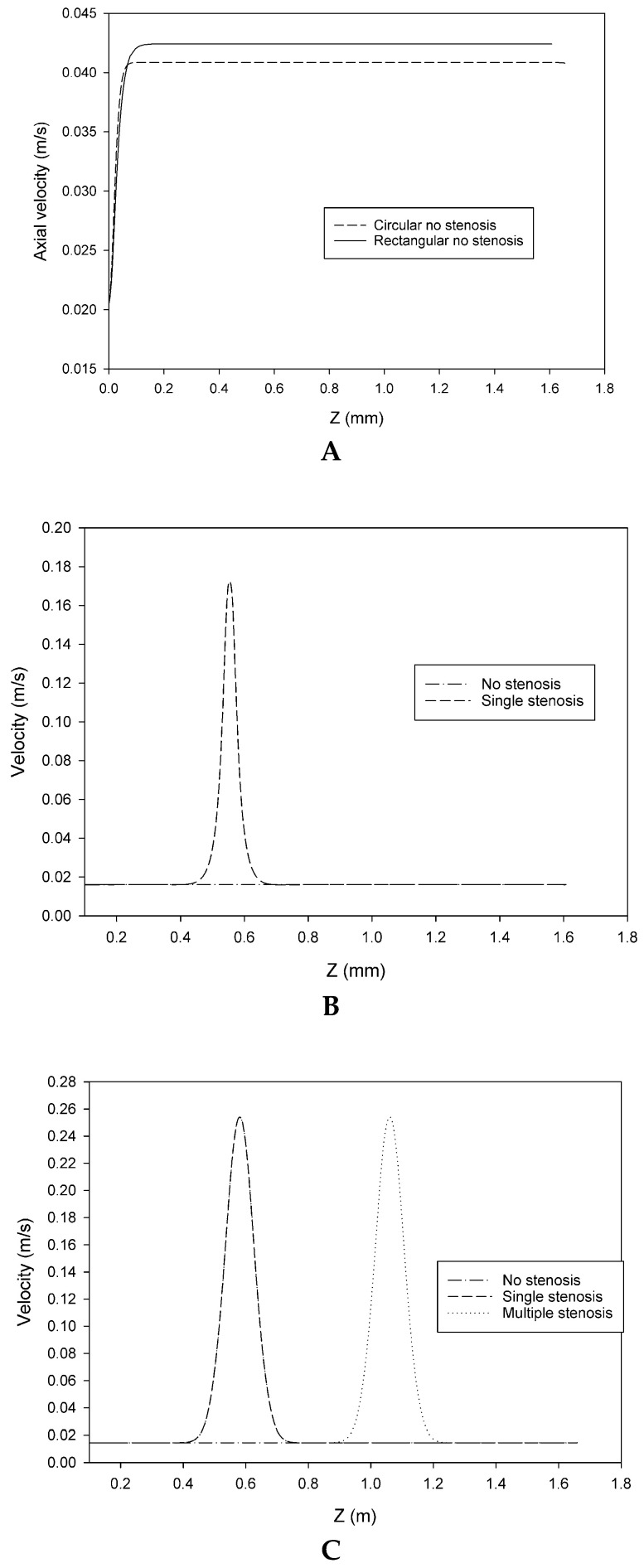
(**A**) Axial velocity profiles along the centerline of the channel for the rectangular and circular microchannels without stenosis. (**B**) Axial velocity profiles along the centerline of the channel for the rectangular microchannel with no stenosis and with a single asymmetric stenosis. Velocity peaks were observed at the stenotic site. (**C**) Axial velocity profiles in circular microchannels under three conditions: no stenosis, single asymmetric stenosis, and double asymmetric stenosis. Distinct velocity peaks align with stenotic locations.

The effect of channel geometry and stenosis configuration on velocity distribution was investigated using five microchannel models: rectangular and circular models without stenosis, rectangular and circular models with a single asymmetric stenosis, and a circular model with double asymmetric stenosis. Flow characteristics, such as symmetric behavior, acceleration, and flow disturbances after the stenosis, were investigated by examining axial velocity profiles and velocity contours, as shown in Figure 3A–C.

#### 3.1.1. Comparison of Rectangular and Circular Microchannels in the Case of No Stenosis

Figure 3A shows the comparison of geometrical shape by examining axial velocities in the rectangular and circular microchannels without stenosis. When no stenosis is present, the rectangular shape has a slightly higher velocity peak than the circular geometry.

#### 3.1.2. Effect of a Single Stenosis in the Rectangular Microchannels

Figure 3B illustrates the effect of a single asymmetric stenosis in the rectangular channel. A sharp velocity peak at the throat was observed when a single asymmetric stenosis was introduced into the rectangular channel, and then a gradual decrease was seen after the stenosis.

#### 3.1.3. Comparison of a Single Stenosis and Double Stenosis in the Circular Microchannel

Figure 3C presents centerline velocity profiles for circular microchannels in the case of no stenosis, single asymmetric stenosis, and double asymmetric stenosis. The velocity profiles for non-stenosed, single-stenosed, and double-stenosed circular microchannels showed that the velocity remained relatively consistent at approximately 0.02 m/s throughout the entire channel, while no stenosis was considered. In the case of single stenosis, one velocity peak was observed at the site of stenosis, and velocity decreases after the stenosis. When two stenoses were introduced, two velocity peaks occurred at the throat regions.

#### 3.1.4. Velocity Magnitude Contours

**Figure 4:**
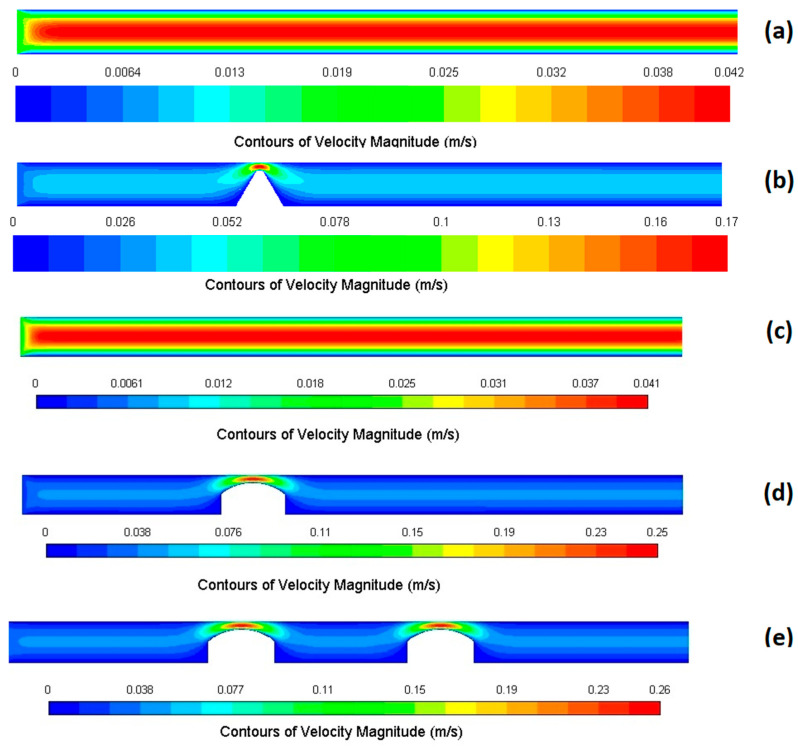
Velocity magnitude contours in the XY plane at the microchannel center for five models: (**a**) rectangular without stenosis, (**b**) rectangular with single stenosis, (**c**) circular without stenosis, (**d**) circular with single stenosis, and (**e**) circular with double stenosis. Asymmetric stenosis introduces significant flow acceleration at the stenotic throat.

Figure 4 shows the velocity magnitude contours in the middle plane for all five cases. Non-stenotic geometries (Figure 4a,c) exhibited similar behaviors, resulting in a spatially uniform distribution along the domain with maximum values occurring at the channel center and smooth decrease towards the walls. Although in the single stenosed models (Figure 4b,d), velocity showed a sharp increase at the throat regions in both rectangular and circular models, they showed different flow patterns. In the rectangular geometry (Figure 4b), the high velocity jet is broader and more extended downstream of the stenosis, and it mostly stays near the centerline. In contrast, a narrower jet, which shifts toward the open side of asymmetric narrowing, is examined in the single stenosis circular channel (Figure 4d). The jet dispersed earlier, which produces a shorter core length in the downstream velocity field because of the curved walls. In the case of double stenosis, two high-velocity jets are observed at the sites of stenosis.

### 3.2. Pressure Distributions

**Figure 5:**
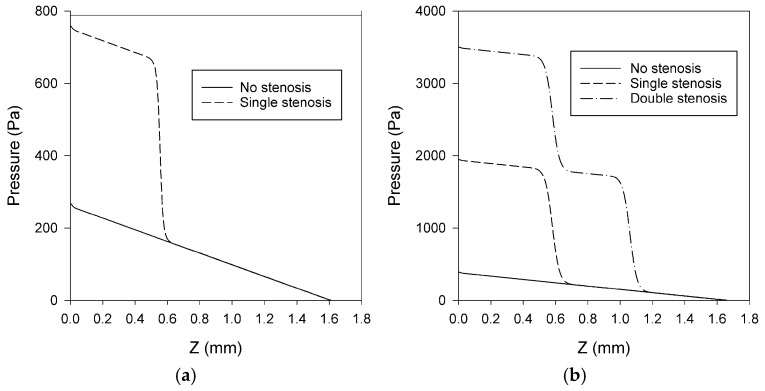
Pressure profiles in (**a**) rectangular microchannels (no stenosis and single asymmetric stenosis) and (**b**) circular microchannels (no stenosis, single, and double asymmetric stenosis). The no-stenosis baseline cases present an almost linear axial drop, but the stenosis induces a step-like ΔP at the restricted area(s), and the case of the double-stenosis model involves two steps.

**Figure 6:**
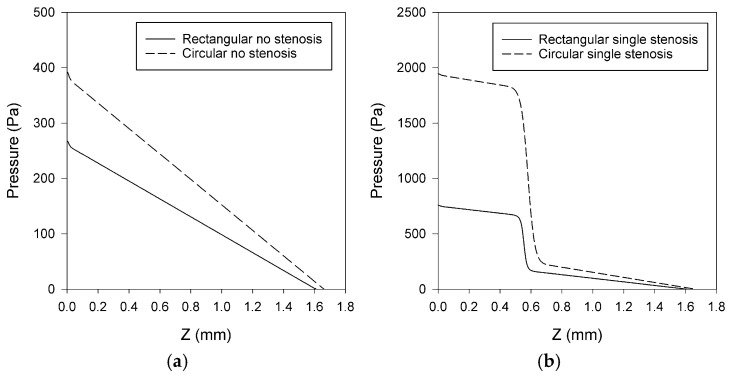
Comparative pressure distributions between rectangular and circular microchannels: (**a**) without stenosis and (**b**) with a single asymmetric stenosis. Circular geometries consistently show larger pressure losses.

**Figure 7:**
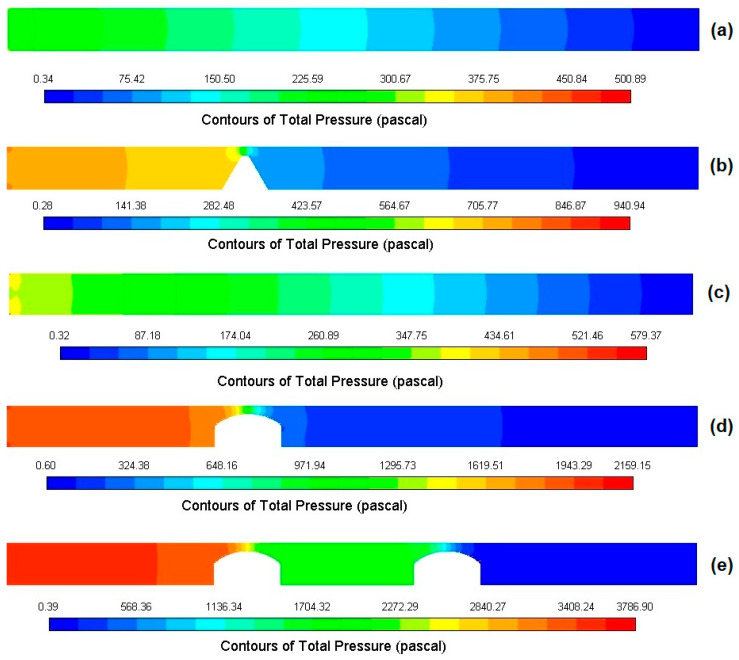
Pressure contours in the XY plane for all modeled configurations: (**a**) rectangular without stenosis, (**b**) rectangular with single stenosis, (**c**) circular without stenosis, (**d**) circular with single stenosis, and (**e**) circular with double stenosis. Pressure peaks and rapid drops are observed at stenotic regions.

Following the velocity analysis, a comprehensive examination of the corresponding pressure alterations in stenosed and non-stenosed microchannels is necessary to gain a more comprehensive understanding of the hemodynamic effects of stenosis. For physiological context, adult systemic arterial pressures are on the order of 80–120 mmHg (≈1.07 × 10^4^–1.60 × 10^4^ Pa). By contrast, the absolute pressures reported below for these idealized, rigid, steady microchannel models are not intended to replicate those physiological values; instead, they are used to compare relative pressure losses across geometries under identical boundary conditions [51]. Figure 5, Figure 6 and Figure 7 show the distribution of pressure in all five models: rectangular channels without stenosis and with a single stenosis, as well as circular channels without stenosis, with single and double stenoses. Since the modeled geometries were idealized microchannels rather than physiological vessels, the reported pressures do not align with physiological blood pressure ranges. They are aimed only for relative comparison among the models. Figure 5 compares rectangular and circular geometries separately, in which the rectangular case includes no stenosis and single stenosis, while the circular case includes no stenosis, single stenosis, and double stenosis. Figure 6 directly compares rectangular and circular microchannels under three conditions: no stenosis, single stenosis, and double stenosis. Finally, Figure 7 illustrates the pressure distributions for all five models, highlighting the effects of both stenosis presence and the number of stenoses.

#### 3.2.1. Pressure Changes in Rectangular and Circular Microchannels

Figure 5 shows that a rapid pressure decrease was observed for both rectangular and circular microchannels in the presence of a single stenosis or double stenosis. In the case of a single asymmetric stenosis, both geometries exhibit a different pressure drop within the stenosis region. While there was one pressure drop for a single stenosis, there were two pressure drops, each corresponding to a separate constriction, resulting in a cumulative pressure loss. To allow scale-independent comparison between rectangular and circular channels under identical inlet velocity and fluid properties, pressure losses were also summarized as dimensionless total loss coefficients (K_tot_). The normalized values preserved the same qualitative trends as the pressure contours: baseline (no-stenosis) losses were higher in circular than in rectangular channels, and asymmetric stenoses introduced strong additional losses, with double > single (Table 5).

Interpreted under fixed-velocity operation, higher ΔP or K_tot_ signifies a design that dissipates more energy at the same operating point, i.e., a device-level attribute rather than an intrinsic shape constant [10,11]. K_local_ isolates the added contraction/expansion penalty of the asymmetric throat(s) within each geometry and should be read as a stenosis-specific contribution on top of the baseline loss [10,11]. These normalized coefficients confirm that the observed differences stem from geometry and stenosis configuration rather than boundary-condition or size mismatches, aligning with the device-relevant comparison point used in this study. Since the inlet velocity was kept constant between the geometries, the rectangular channel operated at 66% higher volumetric flow rate than the circular channel (16.0 vs. 9.66 μL/min; Table 2). Despite the lower flow rate in the circular channel, the total pressure loss was larger, consistent with shape-dependent baseline hydraulic resistance and additional contraction/expansion losses. This device-oriented description under fixed inlet conditions is consistent with common microfluidic practice [5,9], and it also supports previous findings that resistances and local loss coefficients are significantly affected by the cross-sectional shape in laminar microchannels [10]. Because areas and hydraulic diameters are not matched, cross-geometry differences in ΔP and K_tot_ reflect the combined effect of baseline hydraulic resistance and the imposed operating point (fixed U), consistent with this device-oriented framing.

#### 3.2.2. Comparative Effect of Channel Geometry on Pressure

Figure 6 straightforwardly compares the rectangular and circular microchannels. In Figure 6a (no stenosis), the circular microchannel has a higher baseline resistance under non-matched areas and diameters than the rectangular channel at the same inlet velocity as it shows a steeper linear pressure drop. In Figure 6b (single asymmetric stenosis), both geometries present a step-like decline in the constriction, with a more pronounced drop seen in the geometry of the circular case; this corresponds also to further local losses at contraction and expansion. Even when no stenosis exists (Figure 6a), the rectangular channel exhibits a smaller overall pressure drop compared to the circular channel. This suggests that, in terms of hydraulic resistance, geometry plays a significant role, and rectangular channels result in smaller losses than circular channels under the same conditions. In the case of a single stenosis case (Figure 6b), both rectangular and circular channels show a rapid decrease in pressure at the constriction. The rectangular microchannel consistently exhibited a smaller pressure drop compared to the circular channel.

#### 3.2.3. Multiple Stenosis and Distribution Patterns

The effect of stenosis and the number of stenoses are more clearly identified when investigating the pressure distribution across the models. Figure 7 shows the pressure contours along the XY plane for five modeled geometries. It has been observed that the pressure distribution within the channel wall was irregular and segmental. As expected, the pressure at the inlet was consistently greater than at the outlet. Investigating pressure profiles (Figure 5, Figure 6 and Figure 7) in the modeled geometries showed that in a geometry exhibiting a single stenosis, the pressure peak occurs before the stenosis, with a significant decline occurring at the point of narrowing, followed by a plateau or constant pressure profile extending to the remainder of the microchannel. In a model presenting two stenoses, the pressure peaked before the first stenosis and then exhibited a pronounced decline, and then increased again until the second stenosis was reached. A decrease in pressure was observed at the second stenosis, similar to that seen at the first stenosis. When modeling the fluctuating load on channel walls, these fluctuations should be considered, as they could potentially affect flow-induced stresses in blood-contacting devices.

### 3.3. Wall Shear Stress Distributions

**Figure 8:**
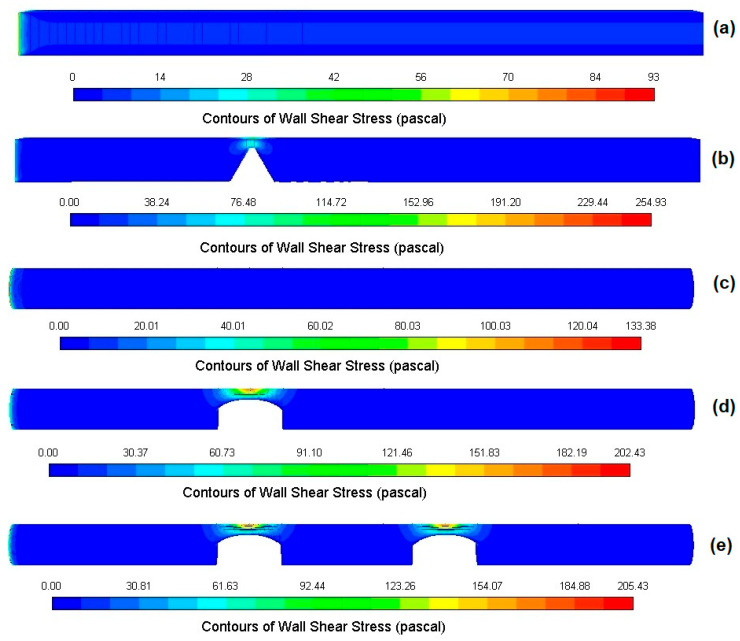
Wall shear stress (WSS) distribution in five geometries with 80% area reduction: (**a**) rectangular without stenosis, (**b**) rectangular with single asymmetric stenosis, (**c**) circular without stenosis, (**d**) circular with single asymmetric stenosis, and (**e**) circular with double asymmetric stenosis. Elevated WSS occurs upstream of each stenosis, with dual peaks in multi-stenotic cases.

In microfluidic thrombosis and hemocompatibility assays, WSS is intentionally controlled over tens to hundreds of pascals to interrogate shear-dependent platelet and vWF responses; the ranges observed here are fully consistent with the high-shear range commonly used in microfluidic experiments [5,8,9]. In such experiments, shear stress is expressed as τ = μ$\dot{\gamma }$ (μ = 0.00345 Pa·s); typical shear rates of 10^3^–5 × 10^4^ s^−1^ correspond to τ ≈ 3–170 Pa, consistent with the peaks reported here [5,8,9,21,28]. Wall shear stress (WSS) is another critical parameter that must be examined following the discussion on pressure fluctuations in microchannels with and without stenosis. Since variations in pressure gradients directly affect the shear forces applied to the channel walls, WSS has significant effects on vascular pathology and physiology. Figure 8 illustrates the WSS distributions for five models with 80% area reductions.

The wall shear stress remained relatively consistent throughout the microchannel without stenosis (Figure 8a,c). Maximum wall shear stress occurred upstream of the throat, thus increasing the probability of plaque rupture at this location. The maximum wall shear stress (WSS) was also determined to be around 200 Pa for the stenosed cases. In the case of double stenosis, the value of WSS exhibited a slight increase compared to that observed in single stenosis, despite the length between the stenosis remaining constant throughout the experiment. The model with single stenosis displayed a single peak, while the model with double stenosis demonstrated two distinct peaks. Elevated WSS and sharp shear gradients are well known to influence platelet and vWF behavior under disturbed flow, as shown in numerous microfluidic and hemodynamic studies [52,53,54]. Consistent with prior hemodynamics literature, elevated WSS and steep shear gradients near asymmetric throats have been shown to trigger vWF-mediated platelet activation and thrombus growth, giving experimental meaning to the simulated WSS peaks observed here [52,53,54].

A summary of peak centerline velocity (U_max_), total pressure drop (ΔP), mean and peak wall shear stress, and the near-wall recirculation length (L_rec_) under identical operating conditions is presented in Table 6. Values were extracted after residual convergence below 10^−6^ using area-weighted surfaces and mid-plane lines, as detailed in Materials and Methods section. Across all five models, no reversed flow was detected along the near-wall line, yielding L_rec_ = 0, i.e., fully attached flow under the present steady-laminar conditions. Dimensionless loss coefficients (K_tot_ and K_local_) are not repeated here to avoid redundancy (see Table 5).

## 4. Discussion

The results of the present study are consistent with the conclusions of prior research, which demonstrated that stenosis significantly impacts flow behavior. This study provides a clear contribution by systematically comparing no-stenosis and single/double asymmetric stenosis in both rectangular microchannels and circular microchannels under the same setup. The findings should be understood as device-oriented responses under fixed operating conditions commonly used in microfluidic devices. They do not constitute a fundamental isolation of cross-sectional shape, which would require matched hydraulic diameters or flow rates. As far as the author’s knowledge is concerned, it provides the first CFD analysis isolating and directly comparing such combined effects of asymmetry and multiplicity in circular microchannels in a cross-geometry comparison. Specifically, stenosis causes increased velocity and wall shear stress (WSS) at the narrowing site, resulting in pressure drops. Understanding the changes in hemodynamics due to asymmetric stenosis is crucial, as it offers basic knowledge about hemodynamic mechanisms.

The underlying mechanisms are as follows. The asymmetry tends to drive the core jet toward the open side of the narrowing. This results in asymmetric velocity peaks and a local rise in wall shear stress at the throat. The channel shape sets the jet width and where it reattaches, which then affects pressure recovery and how far the disturbed-flow region extends. For two asymmetric constrictions, there are two peaks of velocity and WSS, and a cumulative step-like pressure drop with partial recovery between the constrictions.

The results of the present study may have some significant implications for medical device research, as microchannel models can potentially be used as simplified analogs to investigate phenomena associated with flow, which may potentially be relevant in stent and graft development. Given that circular microchannels more closely resemble blood vessels, the observed disparities between rectangular and circular geometries indicate that geometrical considerations should be incorporated into in vitro vascular models to enhance their physiological relevance. The current results also indicate the effects of stenosis severity and inter-stenosis spacing on microchannel hemodynamics. With higher severity, peak WSS, core flow skewing, and near-wall shear heterogeneity can be potentially amplified. On the other hand, greater spacing between consecutive constrictions could result in partial flow recovery, thereby mitigating the effects of downstream shear fluctuations. In future work, these trends will systematically be quantified.

### 4.1. Velocity Field Alterations

The results of velocity profiles and contours showed that both channel geometry and stenosis configuration considerably affect flow behaviors such as peak velocity, jet structure, and flow symmetry. With the help of these findings, mechanisms under different microchannel models can be analyzed in more detail.

When channels with no stenosis are compared, rectangular channels have slightly higher velocity peaks when compared to circular channels. This might be due to the more limited cross-section and flat walls of rectangular shape, resulting in more uniform velocity gradients along the width of the channel. When a single asymmetric stenosis is introduced into the rectangular channel, it can be seen that the velocity decreases asymmetrically downstream of the stenosis, showing a shift in the core flow toward one wall (skewed flow), which can suggest a slight near-wall deceleration along the side opposite to the constriction. However, quantitative velocity analysis along a near-wall line confirmed that the flow direction remained positive throughout, indicating no velocity reversal and therefore no actual recirculation zone (Lrec = 0) under the present steady laminar conditions (Table 6). This behavior can be related to increased shear heterogeneity in stenotic flow. Similar heterogeneity was also observed in the work of Vahidkhah et al. [55], which also highlighted that stenosis can create irregular stress distributions. In all the geometries, there was no reversal in velocity along the near-wall sampling line (L_rec_ = 0), which can be attributed to the extremely small Reynolds numbers and steady inlet conditions in the present study. Under pulsatile stream flows or higher Reynolds number conditions, asymmetric throats can lead to flow separation or intermittency and these will be considered in the upcoming stages of this study.

Rectangular and circular microchannels with single asymmetric stenosis also showed different jet behaviors. The rectangular geometry has a broader jet, which extends downstream of the stenosis and mostly stays near the centerline. Moreover, a more symmetric distribution is observed due to the constriction of lateral momentum because of the flat side walls. In contrast, the circular channel produced a narrower jet, shifting toward the open side of asymmetric narrowing. As can be seen, jet widths after stenosis and alignments are affected by geometrical shape, which affects the formation of shear layers and the extent of disturbed flow regions. These differences, even under identical stenosis severity and location, demonstrate that the wall curvature of different geometrical shapes affects the velocity field, which is ultimately associated with local shear stress patterns and flow recirculations. It was also reported in the work of Zhao et al. [9] that jet width and wall shear stress distributions were controlled by stenosis geometry in microfluidic thrombosis models.

The present velocity fields are laminar because the inlet is steady and the walls are rigid. In real vessels, pulsatile flow and wall motion can strengthen the shear layer near the asymmetric throat and after reattachment. Future work will add pulsatile inflow and compliant walls to check for unsteady features and the onset of instabilities under the same geometry and fluid properties.

The multiple constriction effects can be observed in the double stenosis microfluidic channel, where two distinct velocity peaks are present at the stenosis sites. Although Kabir et al. [36] investigated concentric stenosis rather than asymmetric stenosis, they also observed two peaks at the throat region, indicating that multiple narrowing creates cumulative effects regardless of asymmetry.

### 4.2. Pressure Distribution Alterations

The stepwise pressure drop at the stenotic region can be due to the acceleration at local contraction sites and consequent losses accompanied by the expanded wall loss, leading to additional ΔP and loss of energy. This is as in the classical theory of hydraulic loss in microchannel design; sudden contractions or expansions would cause higher resistance [11].

The classical expectation that a circular duct minimizes hydraulic loss applies to fully developed laminar flow with fixed flow rate and matched cross-section and length. The current models consider a constant inlet velocity, non-matched areas and diameters, and an asymmetric stenosis that implies contraction/expansion losses; so that the total pressure loss is determined by both the baseline-shape effects and local loss coefficient, and the ordering can differ from the classical case. This interpretation corroborates earlier findings of shape-dependent hydraulic resistance in microchannels and the measured inlet and contraction loss coefficients [10,11].

In the double-stenosis situation, consecutive constrictions bring cumulative pressure losses, like the serial loss coefficients reported in the literature for fluidic systems. This has a significant dependence on geometry: the circular channel has a larger peak pressure than the rectangular channel (compare respective Figure 5b with Figure 5a) (on the order of 4000 Pa versus 800 Pa), for which the reason is contrasting hydraulic diameters and baseline resistance values [10]. A possible geometrical meaning of such an effect might be achieved by explaining whether simulations were based on constant flow rate or constant inlet velocity boundary conditions. Additionally, the almost linear axial pressure decrease observed in no-stenosis models is in good agreement with the Poiseuille (laminar) slope, providing a baseline validation of the numerical model. In the case of double stenosis, pressure partly recovered between the stenoses and dropped again immediately after the stenoses, which also supports the cumulative effect of the pressure loss.

Rectangular and circular channels have been compared, showing a typical geometrical contribution towards pressure losses. The rectangular channels generated smaller pressure drops than circular ones in the absence of stenosis, suggesting that they have, in general, lower intrinsic hydraulic resistance for the same flow conditions. This observation is also consistent with theoretical predictions on hydraulic resistance of various cross-sectional geometries, according to which circular cross-sections are generally characterized by larger resistance factors than those of equivalent rectangular channels [10].

In the case of a single induced stenosis, both geometries displayed a sharp step-like decrease at the constriction, but the circular channels continued to experience greater pressure drops. The retention of this ordering indicates that the channel geometry introduces an underlying hydraulic resistance that exaggerates the stenosis. From a device-design standpoint, the lower pressure losses associated with rectangular channels may be exploited to minimize energy dissipation in a microfluidic/blood-contacting device, although this advantage must be balanced against that of fabrication and scaling. Consistently, the dimensionless coefficients (Ktot, and Klocal for stenosed cases) reproduced the same ordering, reinforcing that the higher losses in circular channels and the cumulative effect of double asymmetric stenoses are geometry-driven rather than artifacts of unmatched size or boundary conditions.

### 4.3. Discussion on Wall Shear Stress

The WSS has been widely regarded as a crucial hemodynamic parameter for analyzing stenotic flows [56]. In particular, high WSS and oscillatory WSS have been linked to complex flow fields that may affect wall responses of the vessel [57]. Such an observation was also made in the Lovett et al. [58] and Sweed et al. [59]. In this regard, by investigating WSS changes in simplified microchannel models, an intuitive understanding can be gained as to how stenosis configurations affect the local flow environment.

Concentrated wall shear stress at the throat and steep post-stenotic shear gradients have been linked to fibrous-cap thinning and higher rupture risk in arterial plaques, and to platelet activation in high-shear environments. The WSS ranges reported here therefore provide practical targets for in vitro assays that probe plaque- and platelet-level responses under controlled disturbed flow [52,53].

However, the previous literature has primarily focused on symmetric stenosis (i.e., concentric) [16,30,60], whereas the current study emphasizes the substantial impact of asymmetric stenosis and its resultant asymmetric flow patterns. The work of Kefayati et al. [37] showed that asymmetric stenosis resulted in significant differences in the distribution and magnitude of shear stress, a result that this study also confirms. Moreover, each stenotic region causes a peak in velocity in multiple symmetric stenosis models, as shown in the work of Kabir et al. [36]. Unlike that work, this study complements these results by thoroughly analyzing and comparing single and multiple asymmetric stenosis in different geometric shapes of microchannels, showing how multiple asymmetric stenosis causes large disruptions in flow. The present study corroborates the hypothesis that asymmetric stenosis engenders elevated wall shear stress (WSS) at the stenotic throat. Such a phenomenon has been linked in the literature to a higher likelihood of plaque rupture [58]. A high WSS has been associated with a thinning of the fibrous cap of the atheroma and a higher risk for rupture, being cited as a potential cause of adverse events (such as heart attacks and strokes) [52,53]. In addition to plaque-level effects, increased WSS and high shear gradient effects on cellular mechanobiology have also been demonstrated. For example, high shear stress can lead to platelet activation [53], red blood cell deformation [61], and unfolding of von Willebrand factor (vWF), which has a critical effect on thrombogenesis [54].

It is important to note that while the current study does not actually model cellular reactions, the reported hemodynamic conditions set important boundaries to design microfluidic experiments to explore mechanobiological responses in either stimulated or inhibited disturbed flow. Potential applications are clear. The findings also have the potential to direct the development and standardization of microfluidic devices that mimic disturbed flow for thrombosis and platelet-function testing. They may also aid in screening blood-contacting device concepts under controlled high-shear conditions, and construct more simplistic in vitro vascular tests where shear geometry-driven hemodynamics must be isolated from material and pulsatility. Finally, the comparison of rectangular and circular geometries demonstrates that cross-sectional shape can be used to select baseline resistance and shear exposure in device-scale channels. The potential implications for in vitro device design, these results can be used by selecting circular or rectangular cross-sections to set baseline resistance and exposure to shear. Moreover, the results show that using an asymmetric constriction will localize the wall-shear peaks and the asymmetric jet. Finally, results indicate that when necessary, placing two constrictions will create cumulative pressure losses leading to a dual WSS peak profile with partial recovery in between, which is a useful feature for thrombosis and platelet function assays. These findings suggest that asymmetry and multiplicity can be strategically combined to tune wall shear stress patterns and local shear gradients in microfluidic thrombosis and platelet-activation devices [7,8].

## 5. Conclusions

Asymmetric and double stenosis change flow behavior, which remains inadequately delineated. Essential flow parameters such as velocity, pressure, and wall shear stress (WSS) were comprehensively investigated in rectangular and circular microchannels without and with single and double asymmetric stenosis. The results showed the effect of geometrical shapes and stenotic conditions on axial velocity, shear stress, and fluid acceleration. The hemodynamic fields (velocity, pressure, and wall shear stress) differed among the five models and changed notably in stenosed channels compared with non-stenosed channels.

Constrictions in both rectangular and circular models caused peaks in velocities and maximum WSS values, more evidently in double stenosis models. Asymmetric flow disturbances were examined in asymmetric stenosis, with high values of velocity and WSS at the stenotic throat. With the presence of double stenosis, these effects increased by causing additional pressure drops and greater flow disruptions. These observations show the importance of stenosis configuration and geometrical shapes in causing hemodynamic disruptions. Therefore, these results may be helpful for future studies that investigate the hemodynamics of disease imitation and design vascular devices. Key constraints include rigid walls, steady inlet, a Newtonian fluid, non-matched inlet areas and hydraulic diameters, and fixed shape and spacing of stenosis, all of which represent geometry-based effects but do not account for complete vascular complexity. The observed findings would thus be considered as device-scale responses in simplified microchannels with respect only to certain mechanical properties of stenotic flow, and not fully representative of in vitro vascular models. This work systematically examines for the first time the joint effects of asymmetry and multiplicity at both circular and rectangular microchannels of equal size and stenosis severity under a fixed operating point. Here, these results contribute to a viable, CFD-based design model for channel design and stenosis array selection to construct microfluidic devices to emulate controlled, stenosis-induced disturbed-flow conditions for in vitro hemodynamic and thrombosis studies. In summary, the present results represent device-scale responses under fixed operating conditions. This study does not isolate cross-sectional geometry in the strict sense (areas/diameters/flow rates were not matched); future work will perform matched-Dh or matched-Q comparisons to quantify purely geometric effects. Future work will involve systematic variation in stenosis severity (e.g., from 50% to 90% area reduction) to determine the way in which severity interacts with both geometry and number of stenoses. In addition, a systematic stenosis-to-stenosis spacing study (e.g., one to five diameters) will additionally quantify how the spacing interacts with geometry effects and the effect of the number of stenoses in forming pressure loss, velocity peaks, and wall shear stress. Overall, the results combine the contribution, mechanism, and use cases of asymmetric and multi-stenosis configurations in simple microchannels to offer a reproducible basis for device-oriented in vitro hemodynamics.

## Figures and Tables

**Table 1:** Shear rate values of microchannel models.

Model		<γ> (Volume-Weighted Average, s^−1^)	γ Max (Maximum, s^−1^)
Rectangular	no stenosis	908.86	23,222.68
	single asymmetric stenosis	1076.85	67,881.72
Circular	no stenosis	1099.54	29,729.97
	single asymmetric stenosis	1433.78	56,632.96
	double asymmetric stenosis	1826.13	54,854.73

**Table 2:** Cross-sectional parameters and volumetric flow rates under fixed inlet velocity (U = 0.0205 m/s; ρ = 1060 kg/m^3^; μ = 0.00345 Pa·s).

Geometry	Cross-section (Given)	Area, A (µm^2^)	Hydraulic Diameter, D_h_ (µm)	Inlet Velocity, U (m/s)	Volumetric Flow Rate, Q (µL/min)	Reynolds Number, Re
Rectangular	130 × 100 µm	1.30 × 10^4^	113	0.0205	16	0.71
Circular	D = 100 µm	7.85 × 10^3^	100	0.0205	9.66	0.63

**Table 3:** Mesh convergence results for ΔP and peak WSS between medium and fine meshes.

Case (Same as Figure 2A–C)	Cells (×10^6^) Med → Fine	ΔP Change (%)	Peak WSS Change (%)
(a) Rectangular-single stenosis	0.74 → 0.81	1.47	23.18
(b) Circular-single stenosis	0.27 → 0.87	0.34	1.69
(c) Circular-double stenosis	0.58 → 1.23	0.19	0.05

**Table 4:** Grid Convergence Index (GCI) results for representative cases.

Case	Quantity	r	p	GCI_fine_ (%)	ϕ_ext_ (Pa)	Remark
Rectangular—Single	ΔP	1.035	10.40	4.16	786.6	Monotonic
Circular—Single	ΔP	1.448	1.68	0.48	1958.4	Monotonic
Circular—Double	ΔP	1.286	4.60	0.11	3513.3	Monotonic

**Table 5:** Dimensionless total loss coefficients (K_tot_) and incremental local losses (K_local_) under identical inlet velocity (U = 0.0205 ms^−1^) and fluid properties (ρ = 1060 kgm^−3^).

Geometry	Cases	K_total_	K_local_
Rectangular	No stenosis	1.23	-
Rectangular	No stenosis	1.23	-
Rectangular	Single stenosis	3.42	2.18
Circular	No stenosis	1.80	-
Circular	Single stenosis	8.76	6.96
Circular	Double stenosis	15.8	13.96

**Table 6:** Summary of key flow and wall-shear characteristics for all microchannel configurations operated at the same inlet velocity.

Geometry	Case	Max Velocity (m/s)	ΔP (Pa)	Mean WSS (Pa)	Max WSS (Pa)	Recirculation Length (µm)
Rectangular	No stenosis	0.04242	274.91	4.87	93.46	0 (no recirculation)
Rectangular	Single asymmetric	0.17234	761.37	6.59	290.25	0
Circular	No stenosis	0.04086	400.42	6.04	133.77	0 (no recirculation)
Circular	Single asymmetric	0.24404	1950.95	10.53	202.91	0
Circular	Double asymmetric	0.25418	3510.139	15.23	209.38	0

## Data Availability

The data presented in this study are available on request from the corresponding author due to privacy restrictions.
